# Persistence of Two Non-*Saccharomyces* Yeasts (*Hanseniaspora* and *Starmerella*) in the Cellar

**DOI:** 10.3389/fmicb.2016.00268

**Published:** 2016-03-07

**Authors:** Cédric Grangeteau, Daniel Gerhards, Christian von Wallbrunn, Hervé Alexandre, Sandrine Rousseaux, Michèle Guilloux-Benatier

**Affiliations:** ^1^UMR Procédés Alimentaires et Microbiologiques, Equipe Vin, Aliment, Microbiologie, Stress, AgroSup Dijon – Université de BourgogneDijon, France; ^2^Zentrum für Analytische Chemie und Mikrobiologie, Institut für Mikrobiologie und Biochemie, Hochschule Geisenheim UniversityGeisenheim, Germany

**Keywords:** non-*Saccharomyces* strains, persistence, cellar, *Hanseniaspora*, *Starmerella*

## Abstract

Different genera and/or species of yeasts present on grape berries, in musts and wines are widely described. Nevertheless, the community of non-*Saccharomyces* yeasts present in the cellar is still given little attention. Thus it is not known if the cellar is a real ecological niche for these yeasts or if it is merely a transient habitat for populations brought in by grape berries during the winemaking period. This study focused on three species of non-*Saccharomyces* yeasts commonly encountered during vinification: *Starmerella bacillaris* (synonymy with *Candida zemplinina*), *Hanseniaspora guilliermondii* and *Hanseniaspora uvarum*. More than 1200 isolates were identified at the strain level by FT-IR spectroscopy (207 different FTIR strain pattern). Only a small proportion of non-*Saccharomyces* yeasts present in musts came directly from grape berries for the three species studied. Some strains were found in the must in two consecutive years and some of them were also found in the cellar environment before the arrival of the harvest of second vintage. This study demonstrates for the first time the persistence of non-*Saccharomyces* yeast strains from year to year in the cellar. Sulfur dioxide can affect yeast populations in the must and therefore their persistence in the cellar environment.

## Introduction

Fresh grape berries harbor a wide diversity of non-*Saccharomyces* yeasts (NS). The main genera or species isolated and identified have been (by decreasing order and in relative proportion of the genera/species detected): *Hanseniaspora uvarum*, *Aureobasidium* *pullulans*, *Candida*, *Issatchenkia*, *Metschnikowia*, and *Pichia* (Barata et al., 2012). The population density and diversity of these indigenous yeasts on grape berries are strongly linked to numerous factors such as geographic location, climatic condition, viticultural practices (vineyard management and fungicide treatment), vineyard age, grape variety, sanitary state and berry maturity (Hierro et al., 2006; Xufre et al., 2006; Nisiotou et al., 2007; Barata et al., 2008, 2012; Cadez et al., 2010; Cordero-Bueso et al., 2011a,b; Milanović et al., 2013). An even greater diversity of species has been detected in musts (Jolly et al., 2003) and non-*Saccharomyces* yeast levels can reach 10^6^–10^7^ CFU/ml (Fleet, 2003). The main genera usually found in the first stages of spontaneous fermentation are *Hanseniaspora*, *Candida*, *Metschnikowia*, *Pichia* and, occasionally, *Brettanomyces*, *Issatchenkia*, *Kluyveromyces*, *Rhodotorula Schizosaccharomyces*, *Torulaspora* and *Zygosaccharomyces* (Fleet et al., 1984; Heard and Fleet, 1986; Clemente-Jimenez et al., 2004; Zott et al., 2008; Tristezza et al., 2013; David et al., 2014; Pérez-Martín et al., 2014; Pinto et al., 2015; Wang et al., 2015).

It is now accepted that the yeasts involved in fermentation processes have two possible origins: grapes and the winery/cellar environment (Fleet and Heard, 1993; Mortimer and Polsinelli, 1999). However, the diversity of non-*Saccharomyces* yeast in the cellar has been given little attention. The few studies found in the literature report that the diversity, distribution and percentage of identified species vary depending on the cellar and also depending on the area of the cellar scanned (Sabate et al., 2002; Garijo et al., 2008; Ocón et al., 2010, 2013; Bokulich et al., 2013; Pérez-Martín et al., 2014). Most studies show a higher proportion of NS yeasts in the environment of the cellar compared to the population of *Saccharomyces*. But these percentages vary according to the cellar (Ocón et al., 2010), the different periods of the year (Bokulich et al., 2013; Ocón et al., 2013) and different parts of the cellar (Bokulich et al., 2013). Proportions of NS yeasts reported in the cellar air are variable (Ocón et al., 2013; Pérez-Martín et al., 2014) and high increases in the number and percentage of *Saccharomyces* were observed during the vinification period (Garijo et al., 2008). The main genera in the winery environment (equipment, soil, air) are *Aureobasidium*, *Bullera*, *Candida*, *Cryptococcus*, *Debaryomyces*, *Dekkera*, *Hanseniaspora*, *Kluyveromyces*, *Metschnikowia*, *Pichia*, *Rhodotorula*, *Sporidiobolus*, *Sporobolomyces*, *Torulaspora* and *Williopsis* (Sabate et al., 2002; Sangorrín et al., 2007; Ocón et al., 2010, 2013; Bokulich et al., 2013; Pérez-Martín et al., 2014).

However, the exact role of the winery environment on the microbiota involved in the fermentation, the transfer of yeast communities from the grape berry to the must and the persistence of these yeasts, are poorly understood. The same genus or the same species can be isolated on grape berries, in the must during alcoholic fermentation (AF) and in the winery environment. But at present, it is still difficult to prove the transfer of yeast strains between the vine, the wine and the cellar (soil, air equipment) and their potential persistence over time. To answer this question, it is first necessary to identify the NS yeasts isolated at the strain level and, secondly, to monitor the strains between the different compartments (vine, wine, cellar), as has already been done for *Saccharomyces cerevisiae* strains (Ciani et al., 2004; Le Jeune et al., 2006). Indeed, the existence of a cellar *Saccharomyces* flora has already been demonstrated. Sabate et al. (1998) isolated a large number of *S. cerevisiae* strains common to 2 years during AF. Moreover, the persistence of a commercial *S. cerevisiae* strain in the cellar and its participation in AF 2 years after its last use as a starter were highlighted by Santamaría et al. (2005). Thus some strains seem to persist in the cellar from 1 year to another and could reimplant in grape must during the next vintage. To our knowledge, no monitoring of non-*Saccharomyces* yeast strains has been conducted so far. This study had two objectives: (i) to determine the origin of non-*Saccharomyces* strains isolated in grape must: grape berries and/or cellar, and (ii) to demonstrate their persistence or not in the winery in two consecutive vintages. We selected two yeast genera often found in grape must and implicated in the fermentation process: *Starmerella* reclassified by Duarte et al. (2012) and *Hanseniaspora* for which discrimination at the strain level was possible by Fourier-Transform Infrared (FT-IR) spectroscopy.

## Materials and Methods

### Grape Berry Sampling

Samples of grape berries were taken from a plot of Chardonnay planted in 1986 and located in Burgundy, France (46°18′32.2″N, 4°44′17.9″E, 258 m altitude). The sampling of grape berries or bunches of grapes were carried out six rows of the plot. 18 kg of ripe bunches of grapes were collected aseptically in sterile bags from 60 different vine plants distributed along the six rows (one cluster per plant plant) for the 2012 vintage. Ten berries from each vine plant of each row were collected aseptically for the 2013 vintages (1 kg). Grapes were pressed manually in sterile plastic bags and one sample of must was collected aseptically immediately afterward (noted Tberries). For the 2012 vintage, AF in aseptic conditions at 20°C (2 L erlens) was carried out to enable the development of fermentative genera present but in minority on the bunch. No commercial yeast starter was inoculated in the different musts. Samples corresponding to this enrichment step were noted Tenrich.

### Grape Must Sampling

The harvest was collected manually and placed in 20 kg crates. The 2012 harvest provided 468 kg and the 2013 harvest 100 kg. The must obtained after pressing was left for one night at 10°C, and then distributed into six tanks for 2012 and four tanks for 2013. For each tank, a sample of 50 ml of must was then taken and noted T0 (sample grape must before starting of AF). Immediately after sampling, 30 mg/l of SO_2_ was added in three of the six tanks for the 2012 vintage and in two of the four tanks for the 2013 vintage.

No commercial yeast starter was inoculated in the different musts. AF was monitored by enzymatic dosing of the ethanol produced (Bio-SenTec, France). Samples were taken during fermentation: 3 days after settling (T3), 6 days after settling (T6), 9 days after settling (T9) and at the end of fermentation, (Tf) (data not shown).

### Winery Environment Sampling

For the 2013 vintage, samples were taken from the air, floor and the surface of the winery equipment before the arrival of the harvest. Samples of air (flow rate 100 l/min) were taken using a microbial air sampler, MAS-100 Eco (MBV, Stäfa, Switzerland) placed 1.50 m above the floor. For each sample, a dish with YPD medium (0.5% [w/v] yeast extract, 1% [w/v] peptone, 2% [w/v] glucose and 2% [w/v] agar supplemented with chloramphenicol at 200 ppm to inhibit the development of bacteria) was placed in the air sampler to isolate the yeasts. The volume of air analyzed for each agar gel was 500 l, with three repetitions per sample. A total of 12 samples were taken from the floor and surface of the winery equipment using swabs. After having rubbed the different surfaces by streaking, each swab was placed in a tube containing 1 ml of water supplemented with NaCl (at 0.9% [w/v]) then vortexed for 5 min before analysis.

### Yeast Isolation

Serial dilutions were performed from grape berries and must and 3 × 100 μl of each dilution was spread on the YPD medium described previously and incubated at 28°C. For the samples taken from the winery floor and the surfaces of the winery equipment, 3 × 100 μl of the NaCl solution in which the swab was placed were spread on the YPD medium and incubated at 28°C. For the air samples, the Petri dishes exposed were incubated at 28°C. For all the samples, according to the colonies present, 50 colonies per replicate were selected randomly, purified in YPD medium, then cultivated in liquid YPD medium and finally stored at -80°C in YPD medium supplemented with glycerol (20% [v/v] final concentration).

### Yeast Identification by FT-IR

Identification of yeast isolates was performed by Fourier-Transform Infrared (FT-IR) spectroscopy using a Tensor^TM^ 27 spectrometer coupled with an HTS-XT unit (Bruker, Ettlingen, Germany), as described by Adt et al. (2010), Grangeteau et al. (2015, 2016).

### Strain Typing

Typing of strains belonging to the genera *Hanseniaspora* and *Starmerella* was performed by a hierarchical cluster analysis of the spectra obtained by FT-IR. The dendrogram was calculated using the Average Linkage algorithm and correlation with normalization to reproducibility level. The algorithm is part of the OPUS software package and implemented under the “Cluster analysis” option. The second derivatives of the spectra were used. The frequency ranges were 3,032 cm^-1^ to 2,829 cm^-1^, 1,351 cm^-1^ to 1,200 cm^-1^, and 901 cm^-1^ to 699 cm^-1^. Classification into sub-clusters was done by defining a spectral distance as a value for separation on the strain level. According to Kümmerle et al. (1998) and applied in previous works on strains of the genera *Starmerella* and *Hanseniaspora* (Grangeteau et al., 2015, 2016), all the branches above a spectral distance of 0.3 were sub-clusters, i.e., different strain patterns.

## Results and Discussion

During this study, 4049 yeasts were isolated from grape berries, the cellar environment and musts for the 2012 and 2013 vintages. We focused on two yeast genera often found in grape must and implicated in the fermentation process: *Hanseniaspora* and *Starmerella*. Thus, among these isolates, 214 yeasts were all identified as belonging to the species *Starmerella bacillaris.* 1078 isolates belonged to the genus *Hanseniaspora* of which 313 the species *H. guilliermondii* and 765 were the species *H. uvarum*. In spite of the high number of isolates obtained for these two genera, only two species were identified for the genus *Hanseniaspora* and only one for the genus *Starmerella*. On the contrary, 100 different strain patterns in the 765 isolates belonging to the species *H. uvarum* were identified by FT-IR and for the 313 isolates of the species *H*. *guilliermondii*, 74 different strain patterns were identified by hierarchical cluster analysis of the spectra obtained by FT-IR (Grangeteau et al., 2015). Using this method on FT-IR spectra of 214 isolates of the *S. bacillaris* species, 33 different patterns corresponding to 33 different strains were obtained. This high intraspecific diversity has recently been highlighted for the same species using the microsatellite method (Masneuf-Pomarede et al., 2015).

### Distribution of the Species *Starmerella bacillaris*, *Hanseniaspora guilliermondii*, and *Hanseniaspora uvarum* during the 2012 Vintage

**FIGURE 1:**
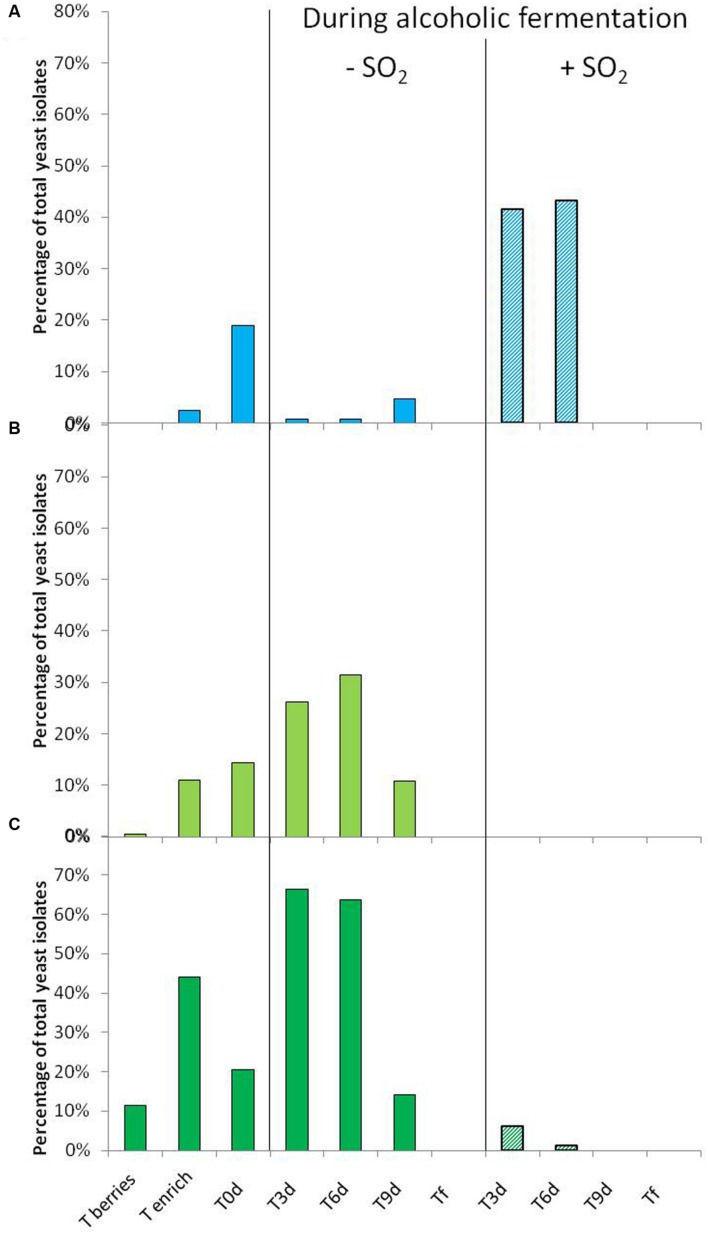
**Percentage of *Starmerella bacillaris* (A), *Hanseniaspora guilliermondii* (B) and *H. uvarum* (C) isolated from berries (T berries, T enrich), grape must (T0d) and during alcoholic fermentation (T3d, T6d, T9d, Tf) without (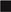) and with SO_2_ (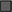) for 2012 vintage.** These percentages correspond to yeast belonging to the respective species compared to the total number of yeast isolates in the corresponding sample time.

The distribution of three species isolated on berries, must and during AF is shown in **Figure 1**. In 2012, the species *S. bacillaris* was not isolated on berries (**Figure 1A**), while the species *H. guilliermondii* was isolated only once (**Figure 1B**) and the species *H. uvarum* represented only 11.5% of total isolates (**Figure 1C**). In spite of the enrichment step, *S. bacillaris* remained proportionally very low (2%) (**Figure 1A**). However, this step allowed isolating a higher number of yeasts belonging to two species of the genus *Hanseniaspora* (11 and 44% for *H. guilliermondii* and *H. uvarum*, respectively). The low presence in particular of *S. bacillaris* and *H*. *guilliermondii* was also observed by Li et al. (2010) for different grape varieties including Chardonnay. Compared to populations isolated on berries, the proportion of these three species isolated in must (T0), obtained after pressing and clarification, was higher: from 0% (berries) to 19% of isolates (must) for *S. bacillaris*, from 0.4 to 14% for *H. guilliermondii* and from 11.5 to 20% for *H. uvarum*. Their presence in must has already been shown in different studies (Xufre et al., 2006; Zott et al., 2008). Several hypotheses may explain the increase in the proportion of these species in must: the selection and modification of the distribution of species linked to changes in environmental conditions such as the modification of osmotic pressure (high concentration of sugars in grape must), pH or available oxygen (Sannino et al., 2013), or enrichment by exogenous yeasts present in the cellar environment (Ocón et al., 2010; Tello et al., 2012). In the absence of SO_2_, during the first days of AF, the proportion of *S. bacillaris* fell considerably compared to T0, while remaining at a low percentage until T9 (1%, 1 and 3% at T3, T6 and T9, respectively) (**Figure 1A**). This species did not appear able to implant itself in the must, which may be explained by the strong presence of the genus *Hanseniaspora* in the same medium during the first days of AF. Indeed, this genus represented more than 90% of the population (**Figures 1B,C**) at T3 and T6, with the strong presence of the species *H. uvarum* (66% at T3 and 64% at T6) (**Figure 1C**). At T9, the proportion of the genus *Hanseniaspora* fell considerably, from 32 to 10% for *H. guilliermondii* and from 64 to 14% for *H. uvarum*. The low presence of *S. bacillaris* when that of the genus *Hanseniaspora* was substantial may result from antagonism between strains, as has already been shown for other yeast strains: between *Brettanomyces* and *Pichia* (Santos et al., 2009), between *Metschnikowia* and *Brettanomyces, Hanseniaspora* and *Pichia* (Oro et al., 2014). None of these three species (*S. bacillaris*, *H. guilliermondii*, and *H. uvarum*) was isolated at the end of AF (Tf). They were replaced by the indigenous species *S. cerevisiae* during AF (data not shown).

In the presence of SO_2_, the behavior observed differed greatly according to yeast genus. During the first days of FA, the proportion of the species *S. bacillaris* increased strongly, continuing until T6 (41% and 46% of the population at T3 and T6, respectively). On the contrary, the proportion of the species *H. uvarum* decreased considerably during the first days of AF (6 and 1% of the population at T3 and T6, respectively) (**Figure 1B**) and the species *H. guilliermondii* was not isolated in the presence of SO_2_ (**Figure 1C**). The implantation of the species *S. bacillaris* appeared to be facilitated following the addition of SO_2_ given its known resistance to this antiseptic (Albertin et al., 2014). Besides its resistance to SO_2_, the rapid and strong development of the species *S. bacillaris* could also have occurred to the detriment of the sparse implantation of the genus *Hanseniaspora*, inhibited by the presence of the antiseptic (Albertin et al., 2014), thereby freeing an ecological niche. Nonetheless, for the three species, the presence of SO_2_ resulted in a rapid decrease in their proportion since they were not found after T9 (**Figure 1**). As for the total disappearance of the species *S. bacillaris* at T9, this may have been directly linked to its sensitivity to ethanol, as shown by Magyar and Tóth (2011). At T9, the content of ethanol in the medium was about 9%v/v whereas it was only 5%v/v at T6. Furthermore, as described by Henick-Kling et al. (1998), the presence of SO_2_ favored the implantation of strains of *S. cerevisiae*, thus leading to faster production of ethanol in the medium. In our study, this implantation of *S. cerevisiae* was observed from T3 and reached 100% of the population at T9 (data not shown), possibly explaining the total disappearance the species *S. bacillaris* at T9.

The results obtained highlight an increase of the population of the three species studied in must compared to that isolated on berry. This increase may be linked either to the implantation of exogenous yeasts or to the preferential development of these species. To verify these hypotheses, the intraspecific biodiversity of the yeasts from grape berries, the grape must and the cellar environment was characterized by FT-IR spectroscopy for the three species studied. This study also allowed highlighting possible differences in resistance to SO_2_ as a function of strain for the three species concerned.

### Intraspecific Study of Populations of *Starmerella bacillaris* in 2012 and 2013

**FIGURE 2:**
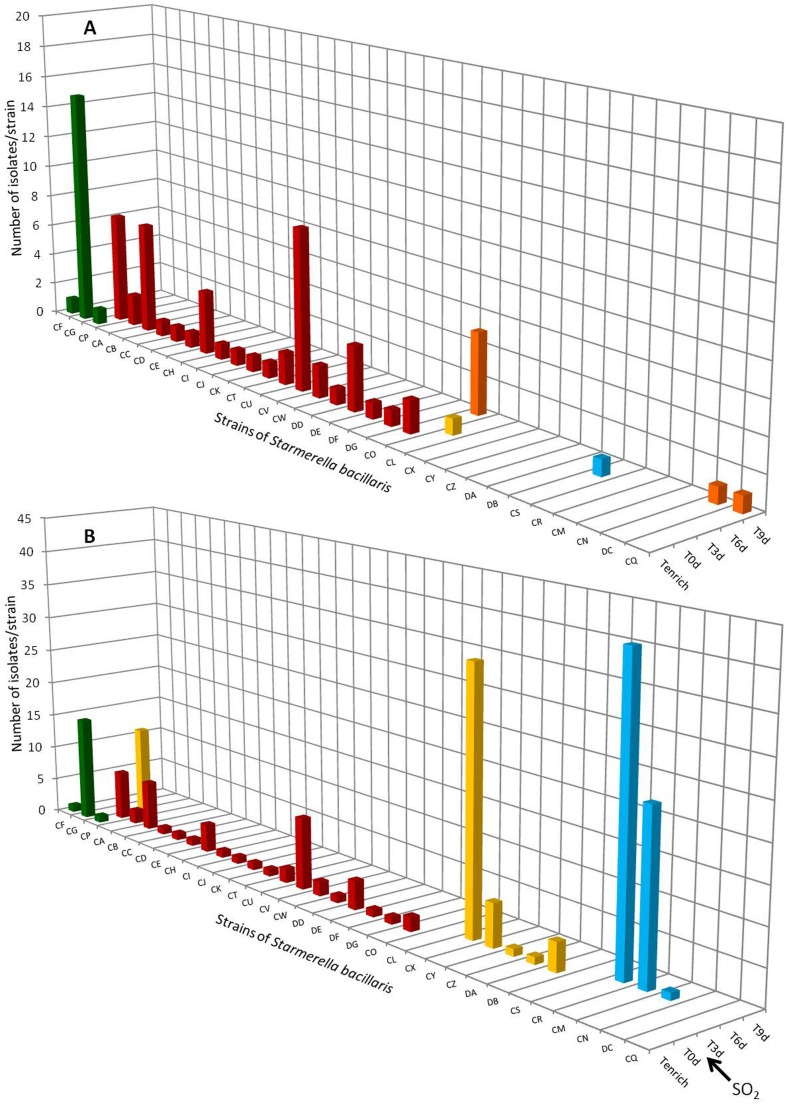
**Distribution of *Starmerella bacillaris* strain patterns during 2012 vintage isolated on berries (T enrich), in grape must (T0d) and during alcoholic fermentation (T3d, T6d, T9d, Tf) without SO_2_ (A) and with SO_2_ (B).** No strain pattern was detected on grape berries without enrichment step.

The results of the intra-specific study of *S. bacillaris* in 2012 are shown in **Figure 2**. The number of strain patterns detected in berries, even following enrichment, was very low (only three different strains: CF, CG and CP). However, high intraspecific diversity was observed in the must (T0, **Figures 2A,B**), since 19 different strain patterns were identified. No strain pattern was seen to be predominant. Only one strain pattern isolated in berries after enrichment (Tenrich) was found in the must at T0, i.e., strain pattern CP. In the absence of SO_2_ (**Figure 2A**), no strain pattern isolated at T0 was isolated again during AF except strain CO isolated at T9. During AF, four new strain patterns (CL, CS, DC, and CQ) were detected but at only one time. These results confirm the low implantation of certain strains of the species *S. bacillaris* during AF. In the presence of SO_2_ (**Figure 2B**), the three different strain patterns isolated on berries after enrichment did not implant during AF, except for strain pattern CP isolated at Tenrich, T0 and T3. The proportion of CP was 4% at T0, before reaching 9% at T3. However, it was no longer isolated afterward (**Figure 2B**). None of the five strain patterns (CO, CL, CS, DC and CQ) isolated during AF without SO_2_ was found in the must fermented with SO_2_. This could be due to the high sensitivity of these strains to SO_2_. On the other hand, in the must fermented with SO_2_, eight new strain patterns were isolated: CX, CY, CZ, DA and DB at T3 and CR, CM and CN at T6. Certain of these strains were present in high percentages of the total yeast population (26% for CX, 28% for CR and 16% for CM). These results highlight for the first time the implantation of strains of *S. bacillaris* stemming from the cellar environment (strains from the air, floor, wine-making equipment and other grape musts fermenting in the winery). The implantation of these eight strains was perhaps aided by the presence of SO_2_ against which their resistance could be higher than the other strains. This implantation could also be due to the disappearance of other strains of *S. bacillaris* and to the disappearance of other yeast species or genera such as *Hanseniaspora* (as mentioned in the results in §3.1). As observed already for *S. cerevisiae* (Vezinhet and Hallet, 1992), the dynamics of the species *S. bacillaris* during AF corresponds to a succession of different strain patterns. Indeed, the five strain patterns isolated at T3 disappeared and then three other strain patterns were isolated at T6 before disappearing too, probably due to the ethanol content of the medium at that time (4–5%v/v) and competition between the yeasts during AF.

The strains found in the must during AF with and without SO_2_ likely originated from the cellar since only one of these strains (CP) was found on the grape berry. In addition, despite the inability of *S. bacillaris* to persist in wine, we wanted to know whether certain strains isolated in 2012 could persist in the cellar environment for 1 year. Thus isolates were obtained before the arrival of the 2013 harvest in the cellar environment (air, floor, equipment). No yeast of this species was isolated. However, *S. bacillaris* had already been found in the cellar environment but in very low proportion and mainly on the type of equipment not used for the fermentations performed in this study (CO_2_ suction line) (Bokulich et al., 2013). This was also the case for the genus *Starmerella* which was not isolated on the grapes harvested in 2013, in the must or during the AF of this vintage. The results therefore appear to indicate the low capacity of *S. bacillaris* strains to persist in the cellar environment from one vintage to the next. However, it is possible that certain strains persisted but that the conditions of the 2013 vintage did not prove propitious for their development and they therefore remained at levels below detection limits.

### Intraspecific Study of Populations of the Genus *Hanseniaspora* in 2012 and 2013

**FIGURE 3:**
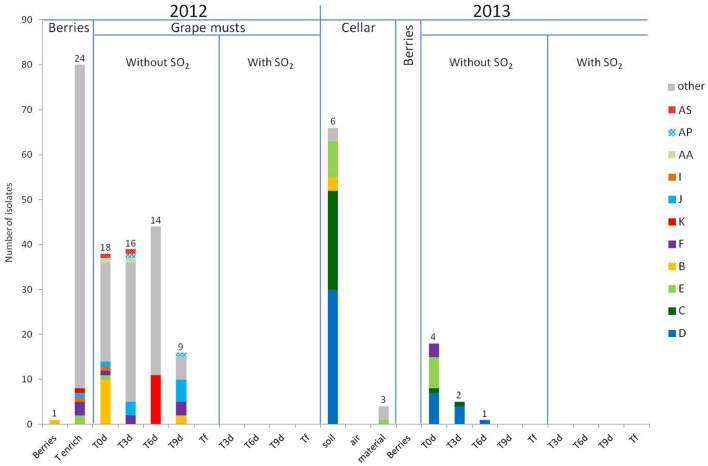
**Repartition of *H. guilliermondii* strain patterns isolated on berries, in grape must, during alcoholic fermentation (T3d, T6d, T9d, Tf) with and without SO_2_ for 2012 and 2013 vintages and in cellar environment in 2013 before the arrival of harvest.** Numbers on the top of the barplot correspond to the number of different strain patterns. The term “other” includes the strain patterns which were detected once.

**FIGURE 4:**
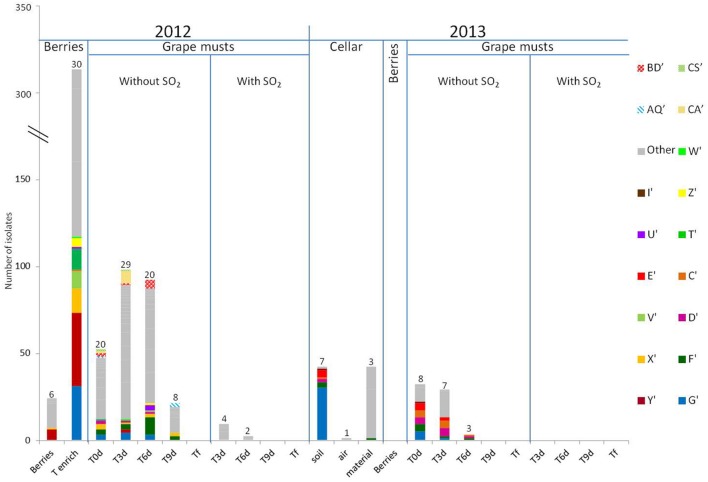
**Repartition of *H. uvarum* strain patterns isolated on berries, in grape must, during alcoholic fermentation (T3d, T6d, T9d, Tf) with and without SO_2_ for 2012 and 2013 vintages and in cellar environment in 2013 before the arrival of the harvest.** Numbers on the top of the barplot correspond to the number of different strain patterns. The term “other” includes the strain patterns which were detected once.

The results of the intraspecific study for *H. guilliermondii* and *H. uvarum* are presented in **Figures 3** and **4**, respectively. In 2012, very considerable diversity was observed for the population of *Hanseniaspora* present on berries following the enrichment step. Thus 24 different strain patterns were isolated for *H. guilliermondii* and 30 for *H. uvarum*. For these two species, the enrichment step proved very useful as it allowed significantly increasing the number of isolates and thus strains isolated (1 strain pattern on berries and 24 after enrichment for *H. guilliermondii*, 6 strain patterns on berries and 30 after enrichment for *H. uvarum*). On the contrary, it is noteworthy that four strain patterns of *H. uvarum* isolated on berries were not found after the enrichment step. Regarding this vintage, and contrary to what was observed for the species *S. bacillaris* (three strain patterns at Tenrich and 19 at T0), the number of different strain patterns was lower in the must at T0: 18 strain patterns for *H. guilliermondii* and 20 patterns for *H. uvarum* compared to the number of strain patterns identified after enrichment: 24 and 30 strain patterns for *H. guilliermondii* and *H. uvarum*, respectively. Of the 24 different strain patterns of *H. guilliermondii* and the 34 different patterns of *H. uvarum* from berries (Tberries and/or Tenrich), only 5 strain patterns (B, E, F, I, and J) for *H. guilliermondii* and three strain patterns (G′, T′, and X′) for *H. uvarum* were found in the grape must at T0. Several other strain patterns present on berries were also detected, not at T0 but during AF. These strain patterns were K (T6) for *H. guilliermondii* and Y′ and W′ present at T3 and U′, V′, and Z′ present at T6 for *H. uvarum*. This confirmed that part of the non-*Saccharomyces* yeasts present in the grape must have come from the vineyard. However, the major part of the strain patterns identified at T0 (13 for *H. guilliermondii* and 17 for *H. uvarum*) and during AF (30 for *H. guilliermondii* and 41 for *H. uvarum*) were not found on the berries and therefore likely came from the cellar environment. The strain patterns found at T0 implanted in the must during the pre-fermentation steps. We can therefore assume that the pressing and clarification steps lead to a selection of strains while favoring the implantation of strains better adapted to grape must conditions. The strains found in the must during fermentation were also certainly better adapted to the medium. In the absence of SO_2_, the number of strain patterns of the species *H. guilliermondii* (**Figure 3**) decreased progressively during AF (18, 16, 14, 9, and 0 strain pattern at T0, T3, T6, T9 and Tf, respectively). Despite this decrease, it is noteworthy that the great majority of strain patterns identified at T3 (12) and all the strain patterns isolated at T6 (14) were not present at T0. Only two strain parttens, AS and AA, were isolated at T0 and at T3 and strain pattern K was isolated at Tenrich and T6. Thus most of the strain patterns found during AF appeared to have originated from the cellar environment. At T9, except for the strain patterns found in very low proportions (5), all the strain patterns present (B, F, and J) have been isolated previously during AF. Thus it appears that from T6, the selection of strain patterns was more related to the increased concentration of ethanol rather than to new implantations of strain patterns. We can observe different cases for these results: strain patterns present on the grape berries (Tberries and/or Tenrich) that persisted during AF (B, F, J, K), strain patterns present on berries and that were found only in the grape must and which did not implant during AF (E, I), strain patterns present on berries and that were never found again (19) and, finally, the large majority of strain patterns (29) probably stemming from the cellar environment and which were isolated once or possibly several times (AA, AP, and AS) during fermentation. Regarding the species *H. uvarum* (**Figure 4**), in 2012 and in the absence of SO_2_, the number of strain patterns increased slightly at the beginning of AF (20 at T0 and 29 at T3). As from T6, the number of strain patterns decreased (20, 8, and 0 at T6, T9 and Tf, respectively). Of the strain patterns isolated throughout fermentation, only AQ′, BD′, CA′, CS′, D′, F′, G′, and X′ were also isolated in the must (T0). This leads to the assumption of strain patterns from outside. Indeed, certain strain patterns not isolated in the grape must at T3 or at T6 were found at T9 (3). Thus there was a succession of strain patterns during AF though much less obvious than that observed for *S. bacillaris.* In the same way as for *H. guilliermondii*, we observed different behaviors of strain patterns of *H. uvarum*: strain patterns present on grape berries (Tberries and/or Tenrich) that persisted during AF (G′, U′, V′, X′, Y′, W′ and Z′), strain patterns present on berries that were only found in grape must and which were not implanted during fermentation (T′), strain patterns present on berries but which were never found again (26), strain patterns probably stemming from the cellar environment isolated in the must (T0) and which persisted during FA (D′, F′, AQ′, BD′, CA′, CS′) or for the great majority of strain patterns (44) that were only isolated once or twice during fermentation.

SO_2_ had a very strong effect on *H. guilliermondii* as this species was no longer detected in the medium after adding this antiseptic (**Figure 3**). These results confirm this species’ low tolerance for SO_2_. Regarding *H. uvarum*, a small number of strain patterns resisted the presence of SO_2_; thus four strain patterns were isolated at T3 and other strain patterns at T6 (**Figure 4**). As with *S. bacillaris*, the strain patterns present during fermentation with SO_2_ were not those that had been isolated at T0. Thus it is likely that the cellar environment contained strains particularly adapted to these fermentation conditions and which implanted and developed following the elimination of less well adapted strains. Resistance to SO_2_ for the species *S. bacillaris* and *H. uvarum* could be strain dependent, as with *S. cerevisiae* (Divol et al., 2006). As observed for *S. bacillaris*, no strain belonging to the genus *Hanseniaspora* (**Figures 3** and **4**) was present at the end of AF whether with or without SO_2_. In 2013, and contrary to *S. bacillaris*, different strain patterns of *H. guilliermondii* (six on the floor and three on the equipment) (**Figure 3**) and *H. uvarum* (seven on the floor, three on the equipment and one in the air) (**Figure 4**) were isolated in the winery before the arrival of the harvest. These results clearly confirm the presence of these species of non-*Saccharomyces* among others in the cellar environment already observed by different authors (Ocón et al., 2010; Bokulich et al., 2013). On the other hand, these results show the presence of different strains of the same species in the cellar environment for the first time. Of these strains in the cellar environment, strain patterns B and E for *H. guilliermondii* and C′, D′, F′, and G′ for *H. uvarum* had already been isolated in 2012. Furthermore, strain patterns B and E (*H. guilliermondii*) and C′ and G′ (*H. uvarum*) came from the vineyard. Also demonstrated for the first time was the capacity of certain strains of *H. guilliermondii* and *H. uvarum* to persist from one vintage to another in the cellar environment. The species *S. cerevisiae* (Sabate et al., 1998; Santamaría et al., 2005) is also known to persist in the same environment, which raises the question whether yeasts of the genus *Hanseniaspora* can implant in musts after staying in the cellar environment for a year in the same way as strains of *S. cerevisiae*.

The most probable source of the *Hanseniaspora* yeasts isolated in the must for this vintage was the cellar environment since no other yeast belonging to the genus *Hanseniaspora* was isolated on berries in 2013. Three strain patterns of *H. guilliermondii* (C, D, and E) (**Figure 3**) and 6 of *H. uvarum* (C′, D′, E′, F′, G′, I′) (**Figure 4**), isolated in musts in 2013 at T0 were found again in the environment before the arrival of the harvest. Among these strain, strain patterns B and E of the species *H. guilliermondii* and D′, F′ and G′ of the species *H. uvarum* had already been isolated in the musts of 2012. They therefore survived for a year in the cellar environment before reimplanting in the musts of the following year. Among the strains that had remained in the cellar environment between 2012 and 2013 only strain pattern B (**Figure 3**) was not isolated in the musts of 2013. These results show the considerable capacity for implantation of these strains after 1 year in the cellar environment. What is more, strain pattern C′ (**Figure 4**) isolated in the vineyard in 2012 but not found again in the musts of 2012 was isolated in the cellar environment before the arrival of the harvest and in the musts in 2013 and at several times (T0, T3, and T6). This strain could have been introduced in the cellar in 2012 with our harvest without having developed sufficiently to be detected. This strain could also have been introduced by the harvests and the later AF performed in the same winery. Lastly, this strain could have been present in the cellar environment during several vintages but not implanted and developed sufficiently to be detected in 2012. This case had already been observed for *Saccharomyces* by Santamaría et al. (2005) who isolated certain strains in one vintage, but not in several succeeding ones, and then found the same strain again. In addition, our results highlighted two strain patterns D′ and F′, not isolated on berries in 2012 but present at every stage of AF (from T0 to T9 for F′ and up to T6 for D′), that persisted in the cellar environment (floor and/or equipment) and which were isolated in the must (T0) and during AF (T3 and T6) in 2013. These strain patterns appeared to be particularly well-adapted to the wine-making environment and the conditions imposed by the wine medium (except for the addition of SO_2_).

Much lower intraspecific diversity was observed for the two species of *Hanseniaspora* in the musts in 2013 (four and eight strain patterns for *H. guilliermondii* and *H. uvarum*, respectively) in comparison to 2012 (18 and 20 strain patterns for *H. guilliermondii* and *H. uvarum*, respectively). This low diversity could be due to the absence of strain patterns stemming from grape and to a lower volume of musts linked to a less abundant harvest in 2013. In the absence of SO_2_, the number of strain patterns of *H. guilliermondii* fell as from the first days of FA. 4 strain patterns were isolated at T0, 2 at T3, 1 at T6 and none from T9. Strain pattern F isolated at T0 was the only pattern not to have been isolated in the cellar environment but which was present in the musts (T0, T3, and T9) and on the berries (Tenrich) in 2012. Regarding the species *H. uvarum*, all the strain patterns except I′, isolated at T0 were still present at T3. At T6, three strain patterns were still isolated and none were from T9 onward. Thus the best adapted strains had undergone selection since the three strain patterns C′, D′ and F′ were present from the start until T6.

In the presence of SO_2_, no strain belonging to the genus *Hanseniaspora* was isolated in the must during fermentation in 2013 whereas strains of the species *H. uvarum* were isolated at T3 and T6 in 2012. Nonetheless, none of the strains that had resisted SO_2_ in 2012 appeared to have subsisted in the cellar environment between 2012 and 2013. Furthermore, several studies have shown that the yeasts of the genus *Hanseniaspora* are quite sensitive to the presence of SO_2_ (Cocolin and Mills, 2003; Albertin et al., 2014). The capacity to resist this antiseptic is undoubtedly a rare characteristic among the strains of the genus *Hanseniaspora*. This was also confirmed in this study by the small number of strains persisting in the presence of SO_2_ in 2012 (only six strain patterns). Lastly, during the 2013 vintage, the species *S. cerevisiae* was present as from T0 whereas it was only detected from T3 in 2012 (data not shown). This initial presence coupled with that of SO_2_ which favors an increase in the proportion of *Saccharomyces* (Henick-Kling et al., 1998) could be detrimental to strains of *Hanseniaspora* in comparison to those of *S. cerevisiae* and explain their disappearance from the beginning of AF (Nissen et al., 2003; Pérez-Nevado et al., 2006).

This is the first time populations of non-*Saccharomyces* yeasts have been studied at the intraspecific level in the vineyard, the cellar environment and grape musts during AF for two consecutive vintages. In spite of the low interspecific diversity for the two genera studied here (a single species for the genus *Starmerella* and 2 for the genus *Hanseniaspora*), high intraspecific diversity was demonstrated for the three species identified: (74 strain patterns for *H. guilliermondii*, 100 strain patterns for *H. uvarum* and 33 strain patterns for *Starmerella bacillaris*). Monitoring these strain patterns in musts during AF showed that, whatever the species considered, there was no really predominant species but rather a succession of different strain patterns, as was observed for the species *S. cerevisiae*.

Furthermore, this study confirmed that using sulfur dioxide eliminates the strains of the genus *Hanseniaspora* and thus permits the development of the species *Starmeralla bacillaris* which is more resistant to this antiseptic. Intraspecific differences regarding resistance to SO_2_ lead, at species level, to the elimination of the most sensitive strains, thereby permitting the development and/or implantation of more resistant strains from the cellar environment.

Lastly, this study showed for the first time the persistence in the cellar environment of strains of non-*Saccharomyces* yeasts capable of reimplantation during the following vintage. Thus, the cellar is not only a transient habitat. However, this capacity is not shared between every yeast species since only two species of the genus *Hanseniaspora* were isolated in the cellar environment during the second vintage. This concerns a limited number of strains: five strain patterns (one for *H. guilliermondii* and four for *H. uvarum*). As described for *S. cerevisiae*, we highlighted for the first time that the non-*Saccharomyce*s flora of the cellar appeared to predominate in comparison to the grape flora. The capacity of species and strains to persist in the cellar therefore influences yeast biodiversity in musts. But an opposite hypothesis could be proposed, namely that yeast biodiversity in must influences the capacity of strains residing in the cellar to implant the must.

## Author Contributions

All authors listed, have made substantial, direct and intellectual contribution to the work, and approved it for publication.

## Conflict of Interest Statement

The authors declare that the research was conducted in the absence of any commercial or financial relationships that could be construed as a potential conflict of interest.
